# The Crucifixion of Dr. Wiley

**Published:** 1912-04

**Authors:** 


THE CRUCIFIXION OF DR. WILEY.—Taft, like Pilate, said: "I find no guilt in this man, His blood be upon you," and they crucified him. The mob of "interests," adulterators and patent medicine fakers and makers said, "Let his blood be upon us and be ——, we are after his scalp and mean to have it," and they got it! Such is the fate of the reformer, always. The gang who fought the pure food bill in Congress and hung it up seventeen years followed up the attack and hounded, vilified and abused Dr. Wiley —made it too hot for him—until he resigned. It is gratifying, however, that he has entered a field where he can perhaps wield a wider influence; he has gone directly to the people. (The people; oh, the people!) That is the one note in politics today, and in the end the people are right. He becomes editor of the Department of Chemistry, etc., in *Good Housekeeping* and will speak to the consumers of food products and users of drugs through that popular magazine. In the end he will triumph—and those who "betrayed" him—like another Judas—it is hoped will have the decency to go off and hang themselves, like he did, for

"Truth is ever on the scaffold; wrong forever on the throne,
Yet that scaffold sways the future, and behind the dim unknown
Standeth God within the shadow, keeping watch above His own."

